# Shedding light on mental health problems and potential solutions for young women: results from an anonymous asynchronous online focus group

**DOI:** 10.3389/fpsyt.2026.1778227

**Published:** 2026-04-10

**Authors:** Renae D. Schmidt, Sophia T. Gonzalez, Sara M. St. George, Chloe A. DiCamillo, Viviana E. Horigian

**Affiliations:** Department of Public Health Sciences, University of Miami Miller School of Medicine, Miami, FL, United States

**Keywords:** mental health, young adults, focus group, social support, loneliness, connectedness, depression, anxiety

## Abstract

**Introduction:**

Mental health challenges - including anxiety, depression, and self-inflicted injury - have risen sharply among teens and young adults in recent years, particularly among females. These trends have unfolded alongside a growing epidemic of loneliness, which has disproportionately affected younger generations. Emerging evidence suggests that social disconnection both exacerbates mental health symptoms and limits access to meaningful social support during critical developmental periods. Although the relationship between social media use and adverse mental health outcomes is well documented, important questions remain regarding the underlying causes of these challenges among young women and how best to assess and address them. This study used asynchronous online focus groups (AOFGs) to explore young women’s perceptions of mental health challenges and to identify strategies to address them.

**Methods:**

Young adult females aged 18–25 living in the United States were recruited via social media platforms to participate in AOFGs in May 2023. Participants completed a baseline survey and anonymously engaged in online discussions by responding to daily prompts and to each other’s posts over three days. Participants were compensated and invited to complete a satisfaction survey. Discussion transcripts were analyzed using thematic analysis.

**Results:**

Fifty-two young adult females participated. Baseline survey results showed high levels of anxiety, depressive symptoms, and social media addiction, alongside low social connectedness. Nine themes were generated via thematic analysis. Several prominent mental health problems were discussed, including depression, anxiety, trauma, loneliness, substance use, and body dysmorphia. Identified contributors included pressures amplified by social media, experiences with misogyny, stigma surrounding mental health, and concerns about the state of the world. Participants expressed the need for connection, trust, healthy conversations, and self-care strategies. They highlighted the need for strong support networks and improved awareness and accessibility of mental health services.

**Discussion:**

Young females are facing significant mental health challenges within a complex social landscape. Addressing these challenges requires strengthening social infrastructure to foster connection, alongside reforming digital environments to support, rather than undermine, meaningful and healing relationships. Future research must develop innovative approaches to reach young women, especially in digital spaces, to address mental health problems at individual, interpersonal, and societal levels.

## 1 Introduction

Young females in the US are facing the largest overall increase in behavioral and mental health problems (1–3), calling the attention of clinicians, researchers, policy-makers, and public health practitioners. Problems center around social media use, anxiety, depression, self-inflicted injuries, and eating disorders, all of which are on the rise among adolescent girls and young adult women (4–9). In fact, alarming statistics highlight that rates of any mental health illness are highest among 18–25 year olds, and are higher among females as compared to males (2). The relationship between social media use and adverse effects on mental health has also been well-documented (10), but not yet fully understood. Loneliness co-occurs with these rising mental health concerns, and may act as a pathway through which social media exposure, internalizing symptoms, and maladaptive coping behaviors intersect and compound risk among young women (11, 12). In fact, the US Surgeon General has underscored the devastating impact of the “loneliness epidemic,” one that is hitting hardest among younger generations. Evidence has demonstrated that social disconnection not only exacerbates mental health symptoms, but erodes access to meaningful social support during critical developmental periods (13). The surge of body dysmorphia is more prevalent in females (14), leading researchers to postulate that body image concerns may be a key mechanism underlying associations between adolescent girls’ and young women’s social media use and mental health problems (15). Further, while drug use trends and rates vary from year to year, data show that nearly one third of 19–30-year-olds reported binge drinking in the past two weeks, and historically high levels of marijuana use, nicotine vaping, and hallucinogens other than LSD (16). Together, these alarming trends underscore the urgent need for action to understand and address the mental health crisis among young women through innovative and targeted interventions.

Mental health is a fundamental component of individual and societal well-being, yet its recognition, definition, funding, and service delivery are shaped by cultural, social, economic, political, and geopolitical forces that influence perceptions, policy priorities, and access to care (17). Therefore, it is critical to contextualize how these trends unfold within a US healthcare system that is largely privatized and insurance-based, characterized by fragmentation, cost barriers, and inequitable access to preventive and early intervention services (18). While the US has the highest rates of mental health diagnoses among high-income countries, it has some of the worst mental health outcomes (19). Countries outperforming the US are ensuring universal, low-cost coverage, investing in equitable primary care and social services, and minimizing administrative burdens (18). Further, mental health service integration has been widely documented in the literature as an effective strategy for identifying and addressing mental health concerns across diverse healthcare settings (20). Nations like the United Kingdom, Australia, Canada, and Scandinavian countries have been leading the way in mental healthcare integration – combining universal or broad coverage models with policies that link mental health care to primary care, employment, and community services, and illustrating more coordinated systems relative to the fragmented, insurance-based approach common in the US (20–22). It is less common in the US to include a mental health provider on primary care teams as compared to countries like Sweden, Australia, and Canada (19). Meanwhile, rates of acute mental health care utilization (e.g., hospitalization and emergency department visits) are higher in the US as compared to international counterparts (23). The US has a relatively low workforce capacity to meet the needs – shortages which have been associated with increased youth suicide rates (24). These structural differences are critical for interpreting perceptions of mental health problems, causes, and solutions in the US.

A current area of concern is the ability to adequately screen, identify, and promptly respond to mental health problems, raising questions about whether the current standardized methods for early identification are sufficient and reliable and whether the approaches are adequately adapted to the needs of Gen Z and Millennial generations. While screening for mental health problems in primary care continues to be the recommendation of the American Academy of Family Physicians (25), and is an important resource and approach, it is challenged by several limitations. First, individuals might see a doctor once a year and may not be inclined to disclose sensitive information (26–28). Second, providers are pressed to screen and evaluate for multiple conditions, limiting the time allotted to mental health problems (29) and third, stigma towards mental health conditions is still prevalent across providers (30, 31). Although scholars have argued contemporary science and clinical practice may be over-pathologizing normative distress among young people, parallel evidence demonstrates that mental health conditions remain under-identified, diagnosed, and treated in healthcare settings (32). These challenges in identification and response highlight the need for more comprehensive and tailored approaches to better understand the mental health crisis among young women.

Recent systematic reviews and meta-analyses on the intersection of social media and mental health symptoms have called for standardized comprehensive behavioral data, including objective assessments of social media use, understanding motivations for use, and more evaluation of the risk and protective factors around use (10, 33). More broadly, among their outlined priorities to transform policy and science to address the mental health crisis facing young people, Gruber and colleagues (34) call for “frequent mental health tracking” and the development of innovative ways to identify, assess, and prevent concerns among these young “digital natives.” These recommendations, alongside the alarming increase in psychosocial concerns among young US females, a fragmented and highly inaccessible US mental health care system, the limitations on early identification, and lacking compatible response that is tailored to young women (35, 36), warrant further investigation as to determining the potential causes of these problems, the best measures to assess symptoms and behaviors, as well as how, when, and where to appropriately identify them. The purpose of this study was to facilitate focus groups with young females to understand their perceptions about mental health concerns among females their age and receive insights on how best to assess and address these concerns.

## 2 Methods

### 2.1 Design

Methods and approach for this study were guided by the members of the Youth Special Interest Group of the National Drug Abuse Treatment Clinical Trials Network (37). Asynchronous online focus groups (AOFGs) were conducted (38), via an online platform wherein participants responded to prompts and other participants’ posts over a series of consecutive days. AOFGs have been identified as an important tool to conduct research on account that they not only allow participants to respond anonymously and from anywhere in their own time, but also harnesses the “…familiarity with and preferences for electronic over face-to-face communication” (39, 40). This approach also has strengths in reaching diverse populations – geographically and otherwise – and may promote a more truthful conversation around sensitive topics (38, 41–43).

For this study, AOFGs were conducted on Discourse.org, a subscription-based, secure online platform which has previously been used for research purposes in other published research studies (44). Participants were asked to respond to two daily prompts over 3 consecutive days. Day 1 included two questions regarding participants’ perceptions of mental health problems most prevalent among females their age, and their root causes (i.e., “What are the main mental health problems among young females your age?” and “What do you think is causing mental health problems among young females your age?”). Day 2 included questions which assessed how and with whom they would like to talk about mental health problems (i.e., “If you had mental health concerns, who would you talk to about it, and what place or platform would you feel most comfortable sharing?” and “What kind of questions would you want someone to ask if they wanted to see how you are?”). Day 3 included questions that explored potential solutions to the problems (i.e., “If you had mental health concerns, what type of help would you like?” and “What do you think would be helpful in regard to mental health problems for young females?”). After responding to the prompts, participants were asked to respond to other participants’ posts to foster engagement and open discussion.

### 2.2 Procedure

Young adult females, 18–25 years old, who spoke English and lived in the US, and had an active email address were recruited via social media. Digital flyers with a description of the study, eligibility criteria, and instructions on participating were posted on Facebook and Instagram (a sample recruitment flyer can be found in **Supplementary Material Figure 1**), leveraging advanced targeting options to reach these specific demographics. Presence or absence of existing mental health symptomatology was not an eligibility criterion. All eligible participants were invited to an online registration portal on Qualtrics to provide informed consent and information including email (for study-related communication), demographic characteristics (age, gender identity, sexual orientation, race, ethnicity), and place of residence. In addition, participants completed a pre-focus group survey with questions about who regularly asks them about mental health concerns, what mental health problems they are asked about, and who they would speak honestly with about these problems. The survey also included validated scales of Generalized Anxiety (GAD-7) (45), Depression (PHQ-9) (46), the Bergen Social Media Addiction Scale (47), the Social Connectedness Scale (48), and questions adapted from the Spirituality Scale (49).

The Qualtrics registration portal utilized several fraud detection features, such as preventing multiple submissions by the same IP address, and the use of Completely Automatic Public Turing test to tell Computers and Humans Apart (CAPTCHA) to ensure that the survey was not being completed by a bot. Upon completion of registration, participants received a copy of their consent form and video instructions to sign in and navigate the study discussion board via Discourse.org. They also received credentials to join one of four pre-assigned online discussion boards (labeled A, B, C, and D), each with 14–15 participants and were assigned a username (i.e. User001A) and password to maintain anonymity. Prior to launching the discussion boards, participants were sent a video which welcomed them to the study and reviewed the values of respect, honesty, engagement, safety, and confidentiality critical for participation in AOFGs.

The discussion boards were active during May 3-May 5, 2023, from 9am to 9pm EST each day. Each morning participants were emailed about prompts going live, and throughout the day every post was immediately reviewed by the study team for safety and confidentiality before being posted to the discussion board. Twice each day, the study team convened to review the discussion boards and reflect on the responses. In some cases, additional questions and prompts were added to the discussion boards to encourage participants to elaborate on their responses and thank them for their sustained engagement. Following the final day of discussion, participants were contacted to complete a brief post-focus group satisfaction survey and were given a list of national mental health resources. Participants were compensated with a $20 Amazon e-gift card per day of engagement where they responded to the main prompts and interacted with a minimum of two peer posts; they received a total of $60 for participating all three days. This study was approved by the University of Miami Institutional Review Board.

### 2.3 Analysis

Frequencies and mean scores were computed for participant baseline characteristics, including demographic information and baseline assessments. Pearson correlation coefficients were calculated to review associations between baseline depressive symptoms, anxiety, social connectedness, and social media addiction.

As for qualitative methodology, each of the 24 transcripts (responses from four groups to two questions per day over three days) was uploaded to NVivo (Version 14) (50) for analysis using the thematic analysis (coding reliability) process described by Braun and Clarke (51, 52). First, two authors (RDS, STG) independently reviewed the data to generate initial codes and collaboratively created a final codebook (**Supplementary Material Table 1**). All data items within the transcripts were independently coded and discrepancies were resolved via a process of consensus. The coders achieved a 72.7% agreement. Four co-authors (RDS, STG, CAD, VEH) then reviewed the coded content, and discussed the content, frequency, co-coding, and interpretation of each code. Finally, the codes were organized into themes that captured common phenomena among the data. These themes were organized around the research aims and related participant questions.

## 3 Results

A total of 52 young women participated in the discussion boards, with 44 (84.6%) participating all three days, 3 (5.8%) participating two days, and 5 (9.6%) participating just one day. The average age of participants was 21.6 (SD = 2.1) with a minimum age of 18 and a maximum of 25. There were 50 participants (96.2%) who identified as cisgender, while 1 identified as genderqueer/gender non-confirming and 1 preferred not to answer. Sexual orientation ranged from 69.2% heterosexual, 19.2% bisexual, 5.8% lesbian, 3.9% asexual, and 2% questioning. The group was comprised of 46.2% White individuals, 34.6% Black/African American individuals, 7.7% Asian individuals, 2% American Indian/Alaskan Native individuals, and 9.6% Multiracial individuals. Regarding ethnicity, 84.6% identified as non-Hispanic, 9.6% as Hispanic, and 5.8% preferred not to answer. The geographic residence of participants covered 19 states, including Alabama, Arizona, California, Connecticut, Florida, Illinois, Indiana, Maryland, Maine, Michigan, Minnesota, Missouri, North Carolina, North Dakota, Nevada, New York, Pennsylvania, Texas, and Virginia.

Several questions and assessments were captured at baseline to characterize the group of participants. When asked if they are ever asked about their mental health, 90.4% of participants said *yes*, and when asked how frequently they were asked about it, 46.8% said *more than once a month*, 23.4% said *once a month* and 17.0% said *once every six months*. When asked about which symptoms or behaviors they were asked about, the most common responses included *anxiety* at 73.1% and *depression* at 63.5% followed by *alcohol u*se (21.1%), *cannabis use* (15.4%), and *tobacco use* (11.5%). Many said that they were asked about mental health by *parents* (63.5%), *friends* (48.1%), *doctor* (32.7%), *counselor* (30.8%), or *family member other than their parent* (21.2%). When asked who they felt they could be completely honest with about mental health concerns, most participants (51.9%) stated they could be *completely honest* with a counselor while 36.5% could be *completely honest* with a doctor, 34.6% with a friend, and 15.4% with a parent. Among participants with severe anxiety or severe depression, none indicated they could be *completely honest* with their parent, but some said they could be *completely honest* with a friend, 34.8% with a doctor (34.8% with severe anxiety; 21% with severe depression), and counselor (65.2% with severe anxiety).

**Figure 1:**
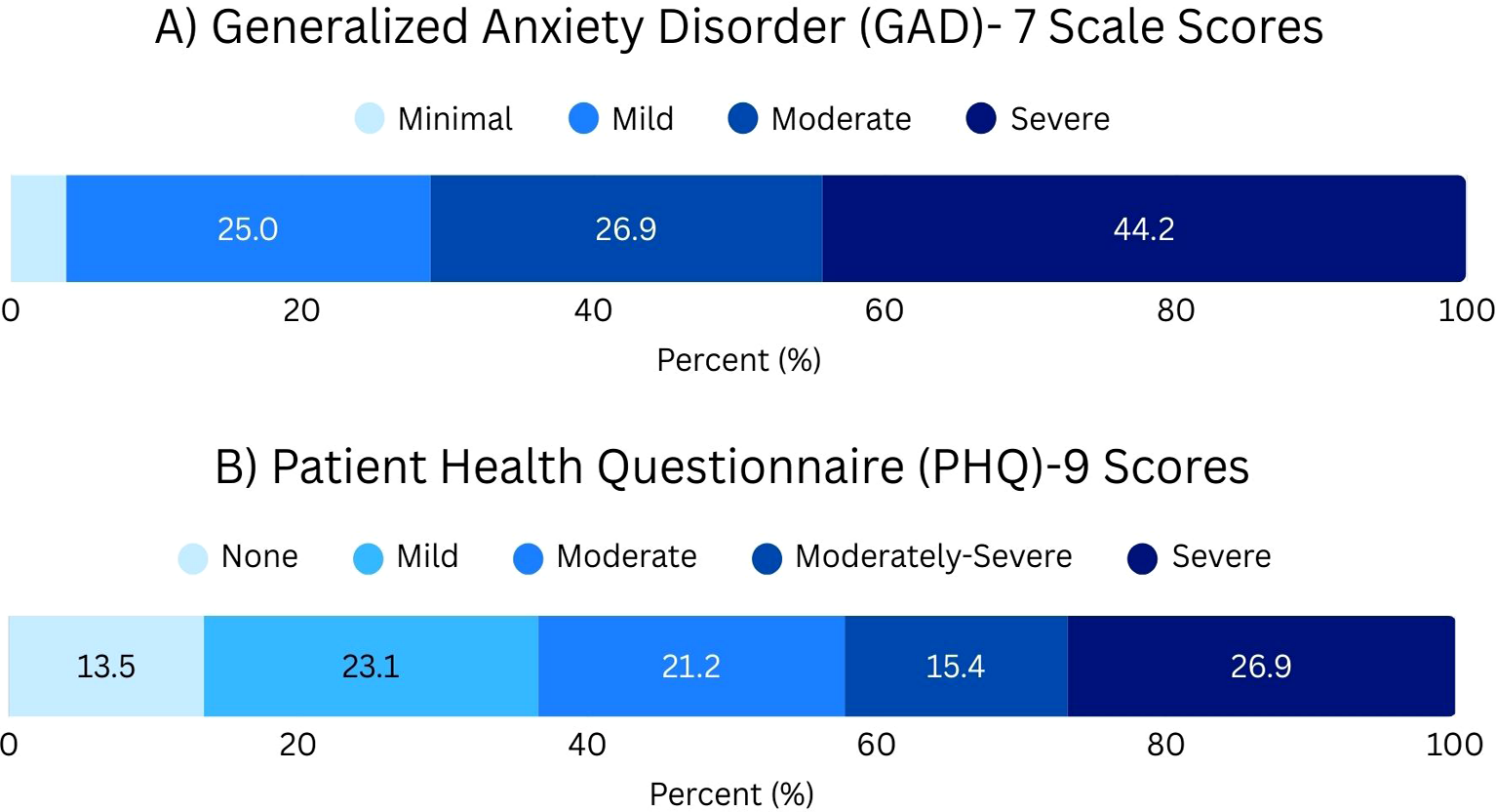
**(A)** Participant GAD-7 scores ranging from Minimal- Severe. **(B)** Participant PHQ-9 scores for participants ranging from None-Severe.

**Figure 2:**
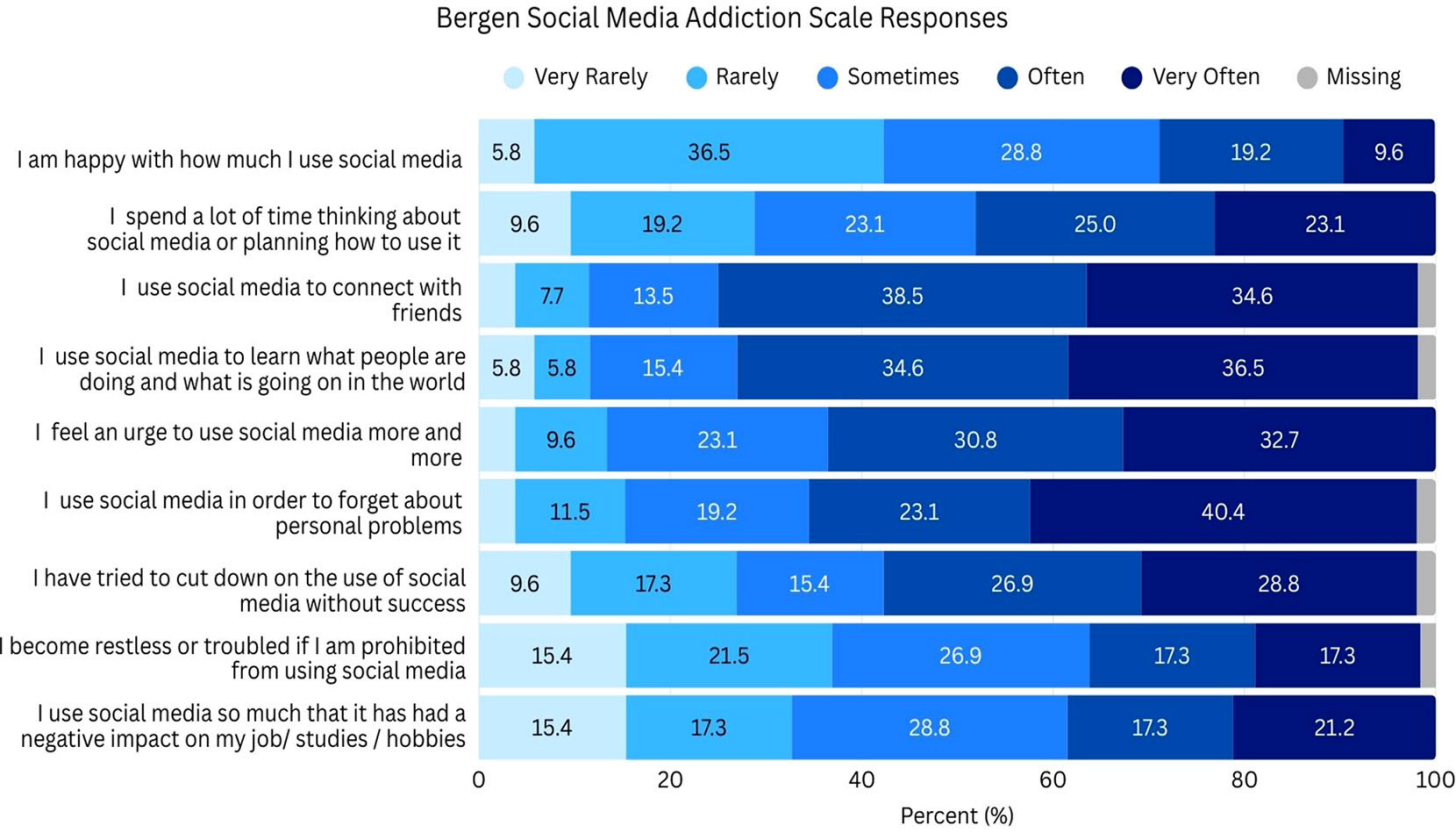
Participant Bergen Social Media Addiction Scale responses with response options ranging from “Very Rarely” to “Very Often”.

Alarmingly, 96.2% participants scored in the categories of Mild (25.0%), Moderate (26.9%), or Severe anxiety (44.2%) on the GAD-7 (**Figure 1A**). Similarly, only 13.5% had no depressive symptoms on the PHQ-9, while most participants fell between Moderate (21.2%), Moderately-Severe (15.4%), and Severe (26.9%) depressive symptoms (**Figure 1B**). The mean score on the Social Connectedness Scale was 49.6 (SD = 15.0) out of 90. The mean score on the Bergen Social Media Addiction Scale was 20.3 (SD = 5.8) out of 30. Responses to items showcased a high proportion of participants indicated *Often* or *Very often* to prompts like “I use social media in order to forget about personal problems” (40.4% *Very often*, 23.1% *Often*) and “I feel an urge to use social media more and more” (32.7% *Very often*, 30.8% *Often*), and “I have tried to cut down on the use of social media without success” (26.9% *Very often*, 28.8% *Often*) (**Figure 2**).

The GAD-7 and PHQ-9 scores were positively correlated (r=0.82714, p<.0001), the GAD-7 score was positively correlated with social media addiction (r=0.64376, p<.0001) and negatively correlated with connectedness (r=-0.61283 p<.0001). Similarly, the PHQ-9 score was positively correlated with social media addiction (r=0.59615 p<.0001) and negatively correlated with connectedness (r=-0.78052 p<.0001).

**Figure 3:**
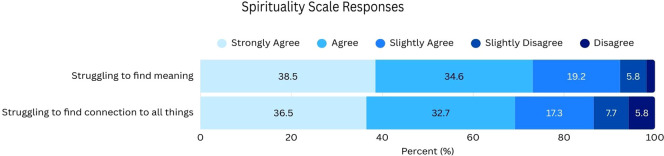
Participant responses to the Spirituality Scale with response options ranging from “Strongly Agree” to “Disagree”.

When asked two prompts about their spirituality among females their age, a large majority either *Agreed* or *Strongly agreed* that females were “…struggling in finding meaning in their lives” (38.5% *Strongly agree*, 34.6% *Agree*), and “…struggling in finding a connection to all things” (36.5% *Strongly agree*, 32.7% *Agree*) (**Figure 3**).

Based on the thematic analysis of participants’ discussion board posts, our research team generated nine themes aligned with research aims and questions to participants, shown in **Table 1**, and described in-depth below.

**Table 1:** Qualitative themes and code frequencies.

Research aims	Participant questions	Themes	Codes (code frequency)
Understand perceptions of mental health concerns among young females	What are the main mental health problems among young females your age?	Several mental health problems are affecting young females, with anxiety and depression at the forefront	Mood Disorders (77) Anxiety Disorders (74) Feeding & eating Disorders (17) Trauma and Stressor-related Disorders (30) Psychotic disorders (3) Obsessive Compulsive Disorders (11) Substance use disorders (10) Self-harm and Suicidality (3) Neurodevelopmental disorders (7) Low self-esteem (27) Mental health problems interfere with life (10) Loneliness and lack of connection (24)
Understand perceptions on the causes of these problems	What do you think is causing mental health problems among young females your age?	Expectations and pressure put on women – often via social media – are damaging	Social expectations & pressure (103) Social media (93) The Female Experience (92)
		The past, present, and future hold trauma, worry, and fear	Adverse Experiences (31) Worry about the state of the world & future (18) The Female Experience (92) Trauma and Stressor-related Disorders (30)
		Part of the problem is not having a voice to talk about it, a place to share, or anyone to listen	Judgment, Stigma, Education, & Awareness (89) Access to care (93) The Female Experience (92) Loneliness and lack of connection (24)
Understand how to assess and who is best to assess these concerns, and what else would help young females if they had mental health concerns	If you had mental health concerns, who would you talk to about it, and what place or platform would you feel most comfortable sharing?	The context of a conversation takes precedence over a specific person or place	Speaking to family friends or friends (69) Role of therapy and speaking to a clinician (98) Importance of support & trust (124) Importance of Healthy conversations (94) Intangible context matters when approaching conversations about mental health (17) Location & modality of help (65) Social media (93) Judgment, Stigma, Education, & Awareness (89)
	What kind of questions would you want someone to ask if they wanted to see how you are doing?	An intentional stance and expressing genuine concern can open the door to an honest conversation; the exact words matter less	General questions (28) Specific questions (26) Introspective questions (4) Asking if they want to talk (6) Offering support, help, & advice (21) Questions about self-care & coping (7)
	What do you think would be helpful in regard to mental health problems for young females?	Self-care can empower females to cope and maintain wellness	Social media (93) Role of self-care (51)
		Individuals and communities should provide essential support	Speaking to family friends or friends (69) Role of therapy and speaking to a clinician (98) Importance of support and trust (124)
		Healthcare system and large-scale societal change are needed	Judgment, Stigma, Education, & Awareness (89) Access to care (93) The Female Experience (92)

### 3.1 Theme 1: several mental health problems are affecting young females, with anxiety and depression at the forefront

Participants across all discussion boards mentioned several mental health problems affecting young females their age. Anxiety and depression were brought up most often and were frequently mentioned together. These were nearly unanimous problems. As one participant put it: “…anxiety and depression take the center stage in people my age.” Another confirmed, “…seems like everyone has [depression] nowadays.” Several other problems were also mentioned less frequently. Participants voiced low self-esteem is a problem they see among young females, especially around physical appearance: “…body image issues have been hugely prevalent for decades for women. It goes so far beyond whether someone is too big or too small, and includes insecurities about hip dips, breast size, stomach rolls, body shapes, and everything else.” Unsurprisingly, participants also mentioned that eating disorders “…seem to be an increasing problem…” One participant shared, “Especially for women, your body is seen as a reflection of yourself. Thinness is considered virtuous, and you should do ANYTHING to attain it, even if it means destroying your body and sacrificing your health.”

Neurodevelopmental issues like ADHD and autism were also discussed, and it was noted that they “tend to go undiagnosed”, especially as they present differently in women versus men. Disorders like OCD and schizophrenia were also mentioned as being “often under-discussed.” It was brought up that some of these rarer conditions face “misconceptions, villainization … and lack of awareness in education [which] prevent us from truly comprehending the capacity to which some people face these mental health issues and their potentially severe effects.” PTSD and stress were also discussed as common problems often leading to other mental health concerns.

“Substance abuse and addiction are also prevalent among young girls…”, one participant shared. It was reflected that young females “may turn to drugs or alcohol to cope with the stress and emotions associated with their experiences.” Another confirmed, “I do think substance use and abuse is on top of the list.” Loneliness was also brought up as “…a huge issue” by one participant, and it was discussed that a general shift to online interaction since COVID-19 has led to people feeling more isolated. Self-harm and suicidality also surfaced. Finally, participants shared how these problems can “…interfere with daily activities and quality of life.” As one participant stated, “…depression can certainly make simply getting up in the morning and going to school each day difficult.”

### 3.2 Theme 2: expectations and pressure put on women – often via social media – are damaging

Participants expressed that societal expectations and pressure put on females are damaging to mental health. They discussed how depictions of influencers on social media can put pressure on females to look, act, and live a certain way. They articulated how influencer culture has driven this aspiration causing disordered perspectives on reality, and that social media leads females to compare themselves to those they see online and warps their perspective on the world. As one participant stated, social media depicts “…contradictory and often unachievable beauty standards…” causing females to have unrealistic expectations about themselves. Participants explained that trends surrounding fashion, beauty, and lifestyle are ever-changing, leading females to chase the next best thing out of fear of feeling inferior. They described how social media is constantly pushing women to be the perfect wives, mothers, and caregivers, while also being independent and successful in their careers. Furthermore, they emphasized that the pressure to meet expectations that are pushed on women via social media can lead to eating disorders, burnout, anxiety, and isolation. As one participant stated, “We’re all burning out with the current state of society. there’s too much pressure.”

### 3.3 Theme 3: the past, present, and future hold trauma, worry, and fear

Participants expressed feelings of continuous worry about the past, present, and future. They shared how past trauma and adverse experiences can be a potential precursor to developing mental health disorders such as anxiety, depression, and PTSD. As one participant stated, “I have been able to recognize that on many occasions, these mental problems echo certain childhood wounds that continue to torment us, because at the time we did not have the tools to manage them, and I think that continues to resonate in our psyches.” Participants reported that they continue to experience trauma, worry, and fear that have been with them since childhood. Moreover, with social media and news outlets at their fingertips, they expressed that the constant influx of bad news causes them to worry about the state of the present and the future. National and global concerns such as a changing economy, reproductive rights for females in the United States, and climate change were common sources of worry and anxiety for participants. One participant stated, “The chances of living a long happy life feel slimmer and slimmer by the year, with economic inequality, conflict, and global warming. Women’s rights are being rolled back across the US. A more virulent strain of pop misogyny has taken hold.” Worrying about one’s financial stability, romantic relationships, and career path were also mentioned as a cause of stress and fear. One participant stated that “depression is a product of our world.”

### 3.4 Theme 4: part of the problem is not having a voice to talk about it, a place to share, or anyone who listens

Participants expressed that part of the problem is not having a voice to talk about mental health, a place to share, or anyone who listens. Many participants disclosed that mental health can be a sensitive topic of conversation, and shared their reservations regarding talking about their mental health. One participant stated, “Even though so many people are struggling with the same things, it can be awkward to talk about.” Some participants clarified that the fear of judgment and the fear of being a burden can keep females from opening up about their mental health. One participant expressed, “I feel that many times I am afraid to express something with my face or see the reaction of other people when I tell them something related to mental health. As if at the bottom of me I felt ashamed for feeling like this, for being human.” Participants voiced concerns about barriers to seeking mental health care such as financial constraints, difficulty navigating the healthcare system, and fear of dismissal of problems by healthcare providers. They expressed a need for guidance on how to find the right resources, navigate the healthcare system, and how to have difficult conversations.

### 3.5 Theme 5: the context of a conversation takes precedence over a specific person or place

While specific types of people were mentioned as those who young females would turn to if they wanted to speak about mental health concerns, ranging from personal contacts including friends, family members, and significant others to clinicians such as therapists and doctors, responses predominantly followed a pattern of emphasizing the *why* behind their choice. Often decisions were based on the sense of comfort and trust they had with them. Some preferred certain individuals over others based on openness and understanding. “My best friends get me better than anybody…”, someone shared. Another participant said they would want to talk to “supportive friends and family who can provide a listening ear and a safe space to talk.” Shared experiences and identities were also criteria lending to the ideal context to share. One participant said, “…since queer therapists of color are so hard to find, it’s much easier to just talk to friends who share my identities.” Other times, participants mentioned the level of severity would drive their decision. One participant explained the rationale behind their choice: “If it was severe, I would like to be able to access a therapist or mental health professional who would help me better address and work though the issues I was experiencing. If it was less severe, I would like a support group or a group of friends where I could express how I was feeling and receive support and feedback.” Participants’ preferences also depend on their overall mood and readiness to share in addition to the relationship with the person. As one participant stated, “I think sometimes I am really in the mood to talk about what is going on and sometimes I’m not.” The participants also voiced that they would be more likely to share when they felt they would not be judged. The importance of support and understanding, as well as active and intentional listening were prominent throughout the conversations across all four groups, pointing to the overarching desire for open, honest conversations with trusted people who care.

Like the types of people, the participants’ rationale for their preferences in location and modality were highly personal and varied from one-on-one conversations to group sessions, and from discussing in-person to posting online (i.e. Reddit, Discord, social media platforms, online support groups). Participant comments about sharing on social media, for example, ranged from positive experiences – “there are threads that are hyper specific to almost everything” – to reasons they would not want to talk about it there – “I wouldn’t talk about it online, I don’t like sharing personal information on social media.” Participants mentioned that sharing specifically anonymously in an online forum feels safer, but also less personal. The opposite was mentioned for in-person conversations, where some participants liked that their privacy was ensured, and it felt more personal, but that it was more difficult to be vulnerable. Rationale for preferring a certain modality or location over other seemed to be based predominantly in levels of comfort and trust. As one participant said, “In terms of where to share, the most comfortable and appropriate place may vary from person to person. Some people may prefer to speak with someone in person, while others may prefer online or remote options, such as teletherapy. It’s important to find a platform that feels safe and supportive for you.”

### 3.6 Theme 6: an intentional stance and expressing genuine concern can open the door to an honest conversation; the exact words matter less

When sharing types of questions they would want to be asked to see how they are doing, participants shared many examples ranging from general to very specific questions. The benefit of broad, general questions is that they can open a space for the respondent to share. For example, one participant noted, “I think I’d honestly like the most simple, straightforward questions like ‘how are you?’ or ‘how have you been feeling?’ or ‘what’s been going on in your life?’ – this keeps things open-ended and lets me talk about however much I feel comfortable sharing.” The benefit of specific questions, as the participants elucidated, is that someone can feel empowered to respond with more than just a brief, default answer. For example, one participant mentioned, “I would like someone to ask me more specific questions about my mental health, for example if I was struggling mentally with school, asking ‘how [did] your biology test go’ instead of just ‘how are you’. I find sometimes when I know a friend is struggling and I just ask ‘how are you’ I only get half of what they really want to say.” Questions about self-care were also shared, for example asking if they have been drinking enough water, sleeping well, and participating in activities they enjoy. One participant stated, “I know in times of stress that my sleep schedule has suffered and I have not been eating like I should. ‘Is there something you can do that relaxes you/distresses you?’ Often when we are upset we are not thinking clearly so suggesting or asking questions like these can help us think of things to help us cope.”

Other questions preferred by participants included ones where the asker is offering support, advice, or simply if they want to talk. For example, participants stated that questions like, “Is there anything on your mind that you’d like to talk about?” and “Is there anything I can do to support you?” can open the door to participants feeling seen and heard, and willing to share. However, while several example questions were given, the focus group discussions especially highlighted the importance of having genuine intentions and that the asker is truly listening. As one participant stated, “The words are less important than the sentiment”. Another said, “I’d just want them to check how I’m doing honestly.” In responses around what would be helpful for mental health problems, participants called for a personal multi-level approach composed of individual (e.g. self-care), interpersonal (e.g. support system), and societal (e.g. destigmatization) components.

### 3.7 Theme 7: self-care is an important means of coping and maintaining wellness

Participants expressed that lifestyle changes and self-care practices could empower young females to improve their mental health. Lifestyle changes such as healthy diet, exercise, adequate sleep, meditation, and mindfulness practices were brought up as essential components to maintaining general wellness and coping with mental health challenges. Additionally, participants emphasized the importance of utilizing educational resources on self-help and practices such as journaling and social media detox. Journaling, participants described, can provide a sense of relief and an avenue where they could articulate and process their emotions as well as brainstorm potential solutions privately. One participant stated “…Journaling helps a lot of the internal thoughts sort themselves, even if it’s to say, “I really need help, I’m noticing a trend of increased depressed thoughts” or “Things have been really terrible lately, circumstantial wise, maybe I need to talk with a friend…” Social media detox was also mentioned as a way to relieve the pressure to stay constantly connected, reduce exposure to harmful content, and open time for in-person connections. Additionally, participants stated that interactive games and apps could help improve consistent engagement in self-care activities. However, although self-care and lifestyle practices were discussed as approaches to help improve mental health, the participants also shared several challenges such as lack of motivation and financial constraints which present barriers.

### 3.8 Theme 8: individuals and communities should provide essential support

Participants stated that a network of people who can provide support and guidance was an essential piece of the puzzle. They shared that although medications and therapy can be helpful, experiencing mental health problems can contribute to feelings of isolation and loneliness. Therefore, they noted that it is essential that they not only have access to treatment but feel seen and supported by a network of individuals. One participant explained, “I don’t want therapy or medications shoved down my throat and feel forgotten about.” The system of support indicated by participants included a variety of individuals encompassing friends, family members, clinicians, support groups, and group therapy. Support networks provide a sense of inclusivity and can foster honest and fruitful conversation, they clarified. In addition, participants expressed the importance of connecting with people who share similar experiences and identities as “…It helps to feel that there are others in the same position and to know you are not alone.” The participants expressed how people who share similar experiences can provide guidance on addressing mental health challenges and where to find useful resources. They also emphasized the importance of having people in their life who were educated on how to best support people experiencing mental health challenges.

### 3.9 Theme 9: healthcare system and large-scale societal change are needed

Participants called for the need to reduce stigmatization of mental health conditions, dismantle toxic expectations of women, and improve the healthcare system. They explained how stigma creates significant barriers when seeking mental health treatment or support from others and emphasized that “Destigmatization would be the number one helpful thing. Being able to seek help in the first place is the only way women are going to get what they need to deal with these issues, so if we create the notion in society that women are not to be shamed for having mental health issues and that they are supported through them, we wouldn’t be so hesitant to seek help…” They called for increased awareness and education on how mental health problems present as well as how to best support family members, friends, and patients. Furthermore, they shared that improving accommodations in workplaces and schools would allow individuals to seek help without affecting their professional or academic careers. Participants called for healthcare system reform and improvements in the accessibility and affordability of mental health care. Importantly, participants expressed the need to dismantle the unrealistic, toxic expectations of women and increase representation of women in the media, government, and healthcare. As one participant recommended, “…diminishing sex appeal in social media/popular news/entertainment I think would put less pressure on women to look/act a certain way (and young men too)!. I think we also need a cultural shift in the way we view a woman’s role in society - for example, eliminating the expectation that women become mothers or are supposed to be primary caregivers.”

## 4 Discussion

This study sought to highlight perceptions of mental health problems among young women and shed light on potential root causes and possible solutions. While not an eligibility criterion, most participants showed high levels of anxiety and depression and many scored high in social media addiction, low in social connectedness, and showed marked need for spiritual connection. Congruent with national reports, participants in the study identified depression and anxiety as prevalent problems but recognized the importance of trauma and other mental conditions requiring attention as well. Participants elucidated several critical causes of these problems, including unachievable expectations, trauma, worry for the future, and challenged mental health services. They shared that meaningfully assessing these problems starts with genuine concern and an intention to truly listen, while approaching them when they are willing and feel comfortable to share. Participants shared that change would be needed at individual, interpersonal, and societal levels - each of which can guide next steps in research and practice.

Aspects of being female or identifying as a woman, including gender role expectations, the effects of misogyny, and the denial of reproductive rights were mentioned as key concerns for participants. They also mentioned how ideals around body image, beauty, and wellness driven by social media, create unrealistic expectations surrounding success and beauty and contribute to constant social comparison and low self-esteem. Bradley and colleagues argued some of the features in social media, such as idealized images of peers and quantifiable feedback, intersect with the importance of peer relationships during development and sociocultural gender socialization processes such as the societal over-emphasis of women’s physical appearance to create the “perfect storm”, exacerbating girls’ body image concerns (15).

Insights on potential solutions and approaches could be organized on individual, interpersonal, and societal levels. At the individual level, participants emphasized the importance of “self-care” as an opportunity for resetting. Several self-care approaches have been described as effective in promoting well-being from being physically active to channeling creativity to practicing mindfulness and meditation (53, 54). Interestingly, participants expressed need to engage in a “social media detox” and presented it as a strategy to reduce the effects of social media and open themselves to in person experiences and connection. A 2025 study found that a 1-week social media detox intervention was significantly associated with reductions in symptoms of depression, anxiety, and insomnia among a sample of primarily young adult women ages 18-24 (55). Findings from this study highlight the influence of social media on mental health and underscore the need for stronger evidence distinguishing healthy from problematic use, as well as co-developed guidance with young women on engaging with social media in healthier ways. At the interpersonal level, participants reflected on the importance of family and friends. Families can play an instrumental role in connecting with and supporting young women’s development by identifying opportunities to open conversations, intentionally listening, and reflecting. Participants recognized mental health stigma and internalized stigma, highlighting the need to educate family and friends on how to navigate difficult conversations and support women with mental health problems. Additionally, when discussing how they would prefer to be asked about mental health problems, what emerged was that an intentional stance and expressing genuine concern is more important than the words, reflecting the importance of empathy, holding, and recognition. Lynch and colleagues (56) note that researchers, practitioners, and policymakers must prioritize the helping relationship in mental health care, emphasizing “trust and confidentiality, supportive rapport, and collaborative treatment” as essential components for delivering effective and meaningful care to young people. One of the most important opportunities to advance and address mental health problems is building strong communities, where people connect, share, and receive and offer help.

Notably, our recommendations align with several key pillars outlined by the Office of the US Surgeon General to combat the loneliness epidemic (57). Specifically, they emphasize strengthening the social infrastructure through community connection and physical spaces to bring people together, cultivating a culture of connection, and reforming the digital environment to ensure that it does not detract from meaningful and healing relationships. Translating these pillars into practice requires that mental health care strategy extends beyond clinical settings to include peer-led spaces, school- and campus-based programming, and community environments that normalize open dialogue and embedded social support. There are opportunities to embed peer navigators in clinical, educational, and community settings, to facilitate peer support spaces both in-person and virtual, to train community members across various diverse settings in mental health literacy and referral pathways, and to integrate digital platforms as bridges to support (58–62). Community-based fitness activities, intergenerational programming, school-based mentorship and service-learning, and companionship phone calls are additional promising approaches highlighted by the US Centers for Disease Control and Prevention (63). Innovative initiatives like Neighbor Day in Australia and Repair Cafes in Europe have shown promise in reducing loneliness (64–66). The KIND Challenge promoting social connection through small acts of kindness to neighbors, also showed potential when tested across the US, UK, and Australia (67). Interventions such as focused brief group therapy (FBGT), initially developed on university campuses, can help young females develop skills and practice new behaviors to help strengthen interpersonal relationships and improve social connection, attending not only to differences in prevalence of symptoms, but also to differences in stress exposure, gender-based discrimination, cultural stigma, help-seeking norms, and experiences with health systems (68). Efforts such as these are instrumental to drive what the Surgeon General termed the “Healing Effects of Social Connection and Community”, for the wellbeing of current and future generations.

At the societal level, access to health care services needs to be expanded and enhanced, from prevention to early identification to treatment. Participants echoed the challenges in accessing health care for mental health problems. Enhancements could include allotting the required provider time for identifying and addressing mental health concerns adequately rather than reacting to and rapidly medicalizing symptoms. Shortcomings in US health care policy – including reimbursement models that prioritize brief visits, fragmented integration of behavioral health into primary care, and limited access to culturally responsive providers – may inadvertently reinforce reactive, medicalized approaches rather than preventive and relational care (18). Current mental health assessment strategies often rely on brief, symptom-focused screening tools administered in time-limited encounters (63). While standardized instruments are informative and have their value, participants’ narratives suggest that these approaches may insufficiently capture the social and gendered stressors shaping their distress. Further, this process requires minimal interaction and may even dismiss real thoughts, feelings, and behaviors. In fact, compared to men and to older counterparts, young women ages 18–25 have been shown to report most frequently that their health symptoms and concerns are dismissed by their health care providers (64). Specific to mental health, one study showed more women than men reported experiencing significant gender bias when receiving care. Providers’ dismissive attitudes were found to be based in notions like linking femininity to emotionality, overlooking women’s mental health issues as hormonal, and neglecting concerns at the intersection of women’s and mental health (65). Clinically, these missed opportunities underscore the need to integrate more contextualized and developmentally attuned assessment practices, including structured time and space for female patients to describe perceived causes of their symptoms and feel genuinely heard, ideally by female physicians as some participants expressed. In practice, this could involve expanding screening protocols to include questions about factors such as social media exposure, societal pressures, geopolitical trauma, and gender-based stress, as well as allocating protected provider time for discussion.

There has been promise of standard primary care partially filling the gap in mental health care at the system-level; however, a 2022 study found participants expressed lower satisfaction with primary care physicians (PCPs) as compared to mental health providers due to reluctance to discuss mental health because of stigma and lack of an established, trusting relationship with their PCP (66). Perceived lack of training, feeling dismissed by their PCP, and a lack of availability for counseling were other reasons mentioned in this study. Structured approaches such as the Collaborative Care Model (67) and other integrated, team-based behavioral health models (68) embed mental health expertise within primary care settings, using measurement-based care and shared caseload management to improve identification, follow-up, and treatment. By redesigning workflows and allocating dedicated team resources for behavioral health, these models are poised to address system-level gaps related to access, fragmentation, and limited specialty capacity.

In addition to integrating behavioral health into primary care, complimentary efforts can be made to expand the accessibility of behavioral health services and meet people where they are. One promising approach is the use of brief, scalable models such as single session interventions which are designed to address mental health concerns in one single encounter as opposed to multiple (69). Singe session therapy has been studied among youth and adults and has been shown to contribute to significant improvements in mental health problems particularly anxiety, panic disorders, and phobias (70). Community-embedded models such as The Friendship Bench program initiated in Zimbabwe trains lay health workers to deliver a 6-session problem-solving therapy intervention to identify and address common mental health problems within informal community spaces (71). Digital innovations have also emerged as alternative pathways to support. Generative artificial intelligence (GenAI) based interventions and wellness applications have demonstrated potential in reducing mental health symptomatology, often anchored in cognitive behavioral therapy techniques and offering anonymous, virtual, and on-demand respondents use GenAI chatbots weekly for mental health issues, many of them due to fear of judgement (72, 73). It should be noted that while they are becoming increasingly popular, ethical concerns are numerous, as general-purpose GenAI chatbots and even many wellness apps are not evidence-based and have shown to produce misinformation, bias, and a misleading sense of therapeutic alliance (74). The appeal is clear though – participants also echoed these sentiments of inaccessible mental health care and the fear of judgment and stigma around seeking help. Efforts to reduce stigma around mental health remain critical for supporting wellbeing and accessible care. A 2025 meta-analysis highlighted how approaches like educational conferences and social contact interventions were significantly associated with attitudes and help-seeking behaviors, though not long-term improvements, pointing to the challenges in sustaining lasting anti-stigma (75). Authors emphasize the importance of integrating lived experience narratives and the opportunities of digital interventions to scale solutions for young people worldwide. They also stress the need for more comprehensive approaches that foster long-term resilience and recovery. Although public discourse has become much more visible in recent years, this does not necessarily equate to the elimination of stigma or equitable access to care. Reluctance to seek help is still common the in US, as are the difficulty and inability to find appropriate treatment (76). Therefore broader societal change includes deepening education and awareness efforts to destigmatize and normalize seeking help for mental health problems – “its ok to not be ok”. Arguably the most challenging steps ahead are the cultural shifts needed to address misogyny, which is still prevalent and harming younger generations of females.

This study has several limitations. First, this is not a nationally representative sample, limiting generalizability. Additionally, we cannot rule out possible sampling bias; recruitment was conducted via social media, and further, individuals who were open to participating in a focus group about mental health may have different opinions and experiences than individuals who did not choose to participate. Second, although the Bergen Social Media Addiction Scale (BSMAS) is a theoretically driven and validated scale (77), this measure may be biased due to self-report and may conflate engagement with social media with addiction (78). Third, while the AOFG methodology has many strengths, there are inherent trade-offs to the approach which can limit the results, including minimal real time opportunity for the research team to ask to follow up questions and less between-participant interaction than if the focus groups were synchronous or held in-person. Finally, while qualitative themes may be influenced by subjective interpretation of the research team, each step of developing and coding themes allowed multiple perspectives and followed a systematic approach. We had acceptable inter-rater reliability and utilized an external auditor and content expert to validate the findings, emphasizing the value of multiple coauthors’ contribution to the data analysis.

The findings of this study highlight first-hand insights into the mental health challenges faced by young females, which are often perpetuated or caused by societal expectations and pressure, sexism and misogyny, and stigmatization of mental health problems. Moving forward, future research must focus on developing innovative and proactive approaches to reach young women, especially in digital spaces, to address root causes of mental health problems at individual, interpersonal, and societal levels. This includes testing and refining current mental health assessment strategies to ensure accuracy, particularly by elevating the voices of those being assessed. Moreover, future research should explore ways to support young women in addressing their mental health concerns by developing and evaluating self-care guidelines, including strategies to mitigate the negative impacts of social media, and providing tools on how to initiate and navigate conversations when seeking help from clinicians or trusted individuals such as family members and friends.

Additionally, research should consider the intersectionality of gender, race, ethnicity, sexual orientation, and other identities in its approaches, attending not only to differences in prevalence of symptoms, but also to differences in stress exposure, gender-based discrimination, cultural stigma, help-seeking norms, and experiences with health systems (68). Importantly, models of care that offer more accessible, comprehensive, and non-judgmental mental health services must be tested. Research should also explore the influence of trusted relationships and community connectedness on help-seeking behaviors and mental well-being to guide effective interventions. Finally, evidence is needed to drive large-scale systemic changes, such as social media reform, promoting community connection, combating misogyny, expanding mental healthcare access, and normalizing conversations around mental health. Only through compassionate, multi-level, and united efforts can we aspire to create a healthier and more resilient future for the next generations.

## Data Availability

The raw data supporting the conclusions of this article will be made available by the authors, without undue reservation.
