# Supplementary material for: Shedding light on mental health problems and potential solutions for young women: results from an anonymous asynchronous online focus group
Source: Front Psychiatry. 2026 Apr 10;17:1778227. doi: 10.3389/fpsyt.2026.1778227 (PMC13106370; doi:10.3389/fpsyt.2026.1778227)
Supplement: Supplementary file 1 [file Image1.pdf]

Supplementary Material - Figure 1. Digital Recruitment Flyer for the LIGHT Research Study

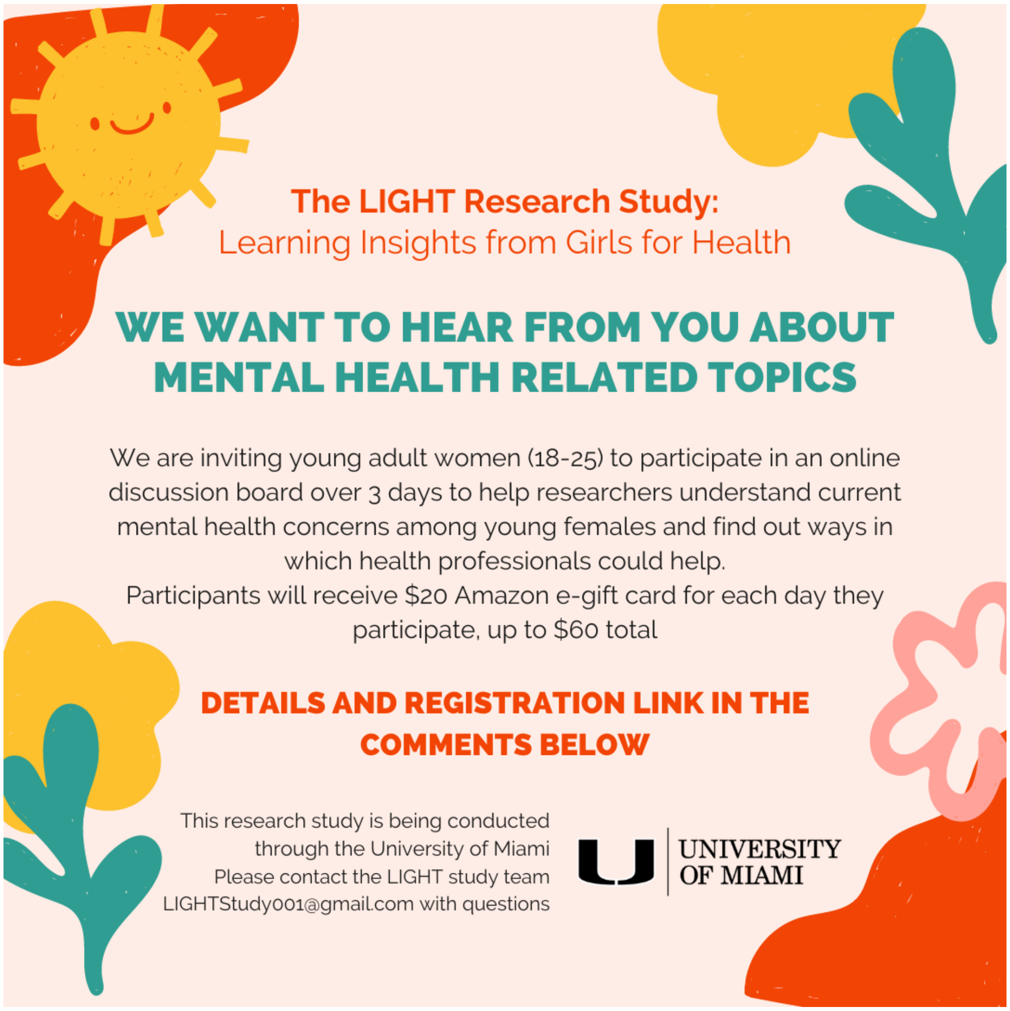

Text posted as a comment alongside digital flyer:

We are inviting young adult women (18-25) to participate in an online discussion board to help researchers understand current mental health concerns among young females and find out ways in which health professionals could help.
You must live in the US, speak English, and have an active email account to be eligible.
Participants will receive up to $60 e-gift card to Amazon.

Step 1: Click the link below to review the consent form, complete a brief baseline survey, and register
Step 2: The study team will email you with video instructions to access the discussion board on Discourse.org
Step 3: Respond to 2 daily prompts and engage with the discussion forum during the 3 consecutive days the board is live
Step 4: Complete a participation satisfaction survey
Step 5: Receive an electronic gift card to Amazon - $20 per each day you participate, up to $60 total if you engage all three days
