# Supplementary material for: Shedding light on mental health problems and potential solutions for young women: results from an anonymous asynchronous online focus group
Source: Front Psychiatry. 2026 Apr 10;17:1778227. doi: 10.3389/fpsyt.2026.1778227 (PMC13106370; doi:10.3389/fpsyt.2026.1778227)
Supplement: Supplementary file 2 [file Table1.docx]

Supplementary Table 1: Codebook

| **Code** | **Description** | **Example Quote(s)** |
| --- | --- | --- |
| Mood Disorders | Participants name depression, Major Depressive Disorder, Bipolar disorder, Postpartum depression | *“… depression is the main mental health problem that I have observed and experienced.”* |
| Anxiety Disorders | Participants name anxiety, phobia, social anxiety, panic attack | *“… anxiety is another big one to think about when girl have a hard time communicating”* |
| Feeding & Eating disorders | Participants name Anorexia, Bulimia, Binge-eating | *“Eating disorders must also be prevalent for those my age, including anorexia and bulimia.”* |
| Trauma and stressor related disorders | Participants name PTSD, Trauma, Acute stress | *“About PTSD, I feel that many times we live it even without recognizing it as a mental disorder. During the last year I experienced this through memories that I kept blocked in my memory for years...”* |
| Psychotic disorders | Participants name Schizophrenia, psychotic disorder | *“… the prevalence of conditions such as OCD, schizophrenia, bipolar and others is often under-discussed. Even though these conditions aren’t quite as common, they are still much more common than some people realize.”* |
| Obsessive compulsive disorders | Participants name OCD, body dysmorphic disorder | *“I have so many close friends who have OCD, bipolar or BPD, and the way these mental illnesses manifest are so different and varied from how they’re portrayed in most media.”* |
| Substance use disorders | Participants name substance use, abuse, addiction, coping using substances | *“Substance abuse and addiction are also prevalent among young girls my age”* |
| Self-harm and Suicidality | Participants name self-harm, cutting, suicidal thoughts or behaviors | *"I used to be a teacher at a middle school and I knew of several cases of self harm among female students, unfortunately. I also observed it myself among peers in high school."* |
| Neurodevelopmental Disorders | Participants name Intellectual disabilities, Communication disorders, ADHD, Autism | *“The main mental health issues among girls my age are probably depression, anxiety, and ADHD… Most girls I know who display symptoms of ADHD still struggle to get a diagnosis and medication, or didn’t receive a diagnosis until their teenage years despite saying they’ve shown symptoms from early childhood.”* |
| Low self-esteem | Participants say a problem is low self-esteem, feeling inadequate, poor self-image | *"… something I have experienced up close is that in addition to being surrounded by a constant bombardment of altered beauty ideals, many times it is also influenced by the adult figures around us. Comments about our weight, how we would look better if we did x or y thing, etc. What accentuates insecurities in us, as well as low self-esteem."* |
| Mental health problems interfere with life | Participants share how mental health concerns have impacted, or could impact, their life including school, work, or other activities | *“Being a highschool student, I feel that many of my female peers struggle mainly with anxiety and depression. From my experience and observations, this makes attending school hard. I have noticed a significant decrease in attendance in many girls I am aware that struggle with these mental health problems."* |
| Loneliness and Lack of connection | Participants say a problem is loneliness, lack of connectedness or support, how no one is checking in | *“Loneliness is a huge issue. Dating apps may help but everyone has different intentions and they might just want your body.”* |
| Social expectations and pressure | Participants explain expectations to fit in a certain social/societal mold, Beauty and wellness Ideals, Social comparison, Pressure to succeed in work, school, and life | *"I think the standards that are put out into the world today for young women are what cause a lot of the health concerns we see. Social media puts out unrealistic standards for beauty and success because most of the time is it fake."* |
| Social media | Participants share perspectives around social media, positive and/or negative | *“I think social media can be complicated because during COVID it was the only way people could stay connected, and it’s often just a way of staying in touch with your friends. But it’s also where we’re exposed to a constant barrage of messaging, some of it deeply harmful.”* |
| The Female Experience | Participants name gender and/or instances where gender seems to matter; Participants point out how being female impacts them: how they are socialized, how their concerns feel unvalidated, their expected roles, experiencing sexism or misogyny, how they are treated in healthcare, hormones, puberty, menstruation | *"Misogyny is definitely a large issue for me personally. I experience extreme paranoia and anxiety around men due to past experiences. It really sucks to feel unsafe at school or work because of misogyny and sexism."   "I think healthcare is such a huge stresser. Not only was Roe v. Wade overturned, but the battle over womens reproductive rights is not over. Pregnancies are not always planned, and laws can get stricted at any moment. Our lives are at stake. And aside from abortion rights, it is so expensive to receive any type of medical care, especially gynecological. If you need medication at any point, you might not be anle to afford it. And therapy is way more expensive."* |
| Adverse experiences | Participants share how negative experiences have caused or could cause mental health problems (violence, harassment, bullying, family conflict, etc.) | *“… mental problems echo certain childhood wounds that continue to torment us, because at the time we did not have the tools to manage them, and I think that continue to resonate in our psyche.”* |
| Worry about the state of the world & future | Participants voice concern about the future, a sense of impending doom due to the state of the world and/or feeling that their future is out of their control, may reference exposure to social media content, politics, climate, etc. | *“With the influx of social media and the ability to have access to some of the most disturbing news, like school shootings or environmental concerns, and being bombarded by it at that, it makes it extremely difficult to keep a level of sanity and to see the “good” that’ll come in the future."* |
| Judgement, Stigma, Education, & Awareness | Participants discuss fear, discomfort, challenges in talking about mental health; OR Participants share a solution to mental health concerns is not feeling judged, being able to speak openly about it | *“I can be hard to talk about struggles and not wanting to feel alienated from your peer by expressing these feelings.”* |
| Access to care | Participants share difficulties or negative experiences in accessing mental health care; OR Participants voice the importance of accommodations and systems set up to help them receive care for mental health concerns | *"Most girls I know who display symptoms of ADHD still struggle to get a diagnosis and medication, or didn’t receive a diagnosis until their teenage years despite saying they’ve shown symptoms from early childhood."*  *"It would be great to have free therapy or counseling to turn to"* |
| Speaking to Family or Friends | Participants state they could talk to their friends, family, significant other | *"… Family, friends or therapist are my first option"* |
| Role of Therapy and Speaking to a clinician | Participants share experiences utilizing therapy to get help for mental health concerns; Participants share they could talk with a therapist/ doctor, may mention specific therapies | *"If I was having a mental health concern, who I discussed it with would vary on the situation. If I felt that it was very serious and significantly affecting my life I would discuss it with my doctor.”* |
| Importance of support and trust | Participants express thoughts or experiences of support to feel included and/or not alone; Participants share that conversations around mental health depend on whether they have a safe, comfortable, established relationship with the person asking | *"The first thing I’d want is support and to not feel alone. A strong support system is always the first step in getting the help you need/want."*  *"Any simple communication helps when someone just wants to feel important. It doesn’t matter the question, no matter how simple, when you just want to feel like someone thinks about you.”* |
| Importance of healthy conversations | Participants share the importance of someone to really listening to them and feeling heard; Participants voice simple, genuine, honest, thoughtful questions or genuine interest and intention can be best | *"Yes totally agreed! People who can tell when to listen and when to talk are so valuable. Also, listen to understand, not to respond. That includes not hijacking stories, not invalidating feelings, and knowing when humor is appropriate and when seriousness is preferred. People don’t always do that maliciously, but it’s a great skill to be a good and empathetic conversationalist."* |
| Intangible context matters approaching conversations about mental health | Participants explain factors like their mood, the overall vibe, and readiness for the conversation are important (more than structural components such as the location or who is asking, or specific questions) | *"This is a hard question because it really depends on the relationship, what kind of mood I’m in, and whether I want to be asked or deal with it on my own."* |
| Location and modality of help | Participants name where they would like to get help, may name online or in person | *"I would also like some type of outlet that I can use to manage my anxiety and depression wether that is some type of app for meditation, journaling, painting etc."  "I agree, I also think speaking with someone in person is the best way for me. I feel more supported when I can see the therapist or other person face to face."* |
| General Questions | Participants name particular general questions or state how general questions are the helpful | *“Are you okay?”  “How have things been going for you lately?”* |
| Specific Questions | Participants state more direct, pointed questions are helpful to not just get a general response | *“Have you been able to participate in activities you enjoy?”  “I would want him/her to ask if I have been sleeping well?”  “Are you finding time for yourself with all these exams coming up?”* |
| Introspective questions | Participants share that helpful questions are those prompting reflection on reasons behind their feelings | *“Why do you think you feel that way?” “What experiences happened to you that made you feel this way?”* |
| Asking if they want to talk | Participants state questions opening an invitation to share more | *“Is there anything on your mind that you’d like to talk about?”  “Do you want to tell me more?”* |
| Offering support, help, & advice | Participants name questions that position the asker to take an active role to be a part of the solution or offer anything | *“What can I do to support you?”*  *“Do you want me to give you some advice, my opinion or did you just want to let it go?”* |
| Questions about self-care & coping | Participants name questions that ask about what they are doing for themselves to manage their physical and/or mental health | *“How are you taking care of yourself?” “How have you been managing stress?”* |
| Role of self-care | Participants discuss self-care, meditation, mindfulness, journaling, working out, and other practices individuals can do on their own | *"Lifestyle changes such as regular exercise, healthy eating habits, and good sleep hygiene."*  *"I agree that writing things down can help! Sometimes the act of articulating what I’m feeling into words helps me process it and makes it easier to handle."* |
